# What Is the Impact of Early and Subsequent Epidemic Characteristics on the Pre-delta COVID-19 Epidemic Size in the United States?

**DOI:** 10.3390/pathogens11050576

**Published:** 2022-05-13

**Authors:** Hao Lai, Yusha Tao, Mingwang Shen, Rui Li, Maosheng Zou, Leilei Zhang, Lei Zhang

**Affiliations:** 1 China-Australia Joint Research Center for Infectious Diseases, School of Public Health, Xi’an Jiaotong University Health Science Center, Xi’an 710061, China; xjtu_haolai@stu.xjtu.edu.cn (H.L.); mingwangshen521@xjtu.edu.cn (M.S.); yananlirui@stu.xjtu.edu.cn (R.L.); zms1965@stu.xjtu.edu.cn (M.Z.); 3120115086@stu.xjtu.edu.cn (L.Z.); 2 SESH (Social Entrepreneurship to Spur Health) Global, University of North Carolina at Chapel Hill Project-China, Guangzhou 510095, China; yusha.tao@seshglobal.org; 3 Key Laboratory for Disease Prevention and Control and Health Promotion of Shaanxi Province, Xi’an 710061, China; 4 Artificial Intelligence and Modelling in Epidemiology Program, Melbourne Sexual Health Centre, Alfred Health, Melbourne, VIC 3053, Australia; 5 Central Clinical School, Faculty of Medicine, Monash University, Melbourne, VIC 3800, Australia; 6 Department of Epidemiology and Biostatistics, College of Public Health, Zhengzhou University, Zhengzhou 450001, China

**Keywords:** COVID-19, pre-delta, epidemic size, early prediction, emerging infectious diseases, United States

## Abstract

It is still uncertain how the epidemic characteristics of COVID-19 in its early phase and subsequent waves contributed to the pre-delta epidemic size in the United States. We identified the early and subsequent characteristics of the COVID-19 epidemic and the correlation between these characteristics and the pre-delta epidemic size. Most (96.1% (49/51)) of the states entered a fast-growing phase before the accumulative number of cases reached (30). The days required for the number of confirmed cases to increase from 30 to 100 was 5.6 (5.1–6.1) days. As of 31 March 2021, all 51 states experienced at least 2 waves of COVID-19 outbreaks, 23.5% (12/51) experienced 3 waves, and 15.7% (8/51) experienced 4 waves, the epidemic size of COVID-19 was 19,275–3,669,048 cases across the states. The pre-delta epidemic size was significantly correlated with the duration from 30 to 100 cases (*p* = 0.003, r = −0.405), the growth rate of the fast-growing phase (*p* = 0.012, r = 0.351), and the peak cases in the subsequent waves (K_1_ (*p* < 0.001, r = 0.794), K_2_ (*p* < 0.001, r = 0.595), K_3_ (*p* < 0.001, r = 0.977), and K_4_ (*p* = 0.002, r = 0.905)). We observed that both early and subsequent epidemic characteristics contribute to the pre-delta epidemic size of COVID-19. This identification is important to the prediction of the emerging viral infectious diseases in the primary stage.

## 1. Introduction

Coronavirus 19 disease (COVID-19) has led to a worldwide pandemic [1,2]. As of 31 March 2021, the pandemic disease was affecting people in over 200 countries and territories, with more than 127 million confirmed cases and 4 million deaths reported globally [3]. At the same time, in the United States (US), there were over 30 million confirmed cases and 550,354 deaths attributed to COVID-19 [4,5]. The cumulative incidence of COVID-19 in the United States at that time exceeded 9000/100,000. Whereas, at that time, only 16% of Americans were completely vaccinated, and the delta variant was just beginning to spread in the United States [6,7,8]. It can be considered that the epidemic of COVID-19 in the United States was in an early stage up to that time. Exploring the factors related to the early epidemic in the United States may provide new knowledge and insights for COVID-19 control and the other emerging viral infectious diseases.

After experiencing the early small-scale spread of the COVID-19 epidemic, many areas in the United States still experienced multiple outbreaks and spatiotemporal changes despite the government’s interventions, such as repeated lockdowns and the wide use of face masks [9,10,11,12]. Previous studies have demonstrated that early epidemiological characteristics, such as the basic reproduction ratio, could determine the subsequent epidemic level [13,14]. Furthermore, early epidemiological characteristics (such as the number of days from 30 to 100 cases) have been hypothesized as significant predictors of the subsequent epidemic of COVID-19 in China [15]. It is still uncertain whether this hypothesis can be confirmed in the United States from the available epidemiological data.

Various mathematical methods have been used to describe and predict the trend of the COVID-19 epidemic in the United States [16,17,18,19], with a lot of critical mathematical indicators produced. However, most studies focused on the prediction by models, as to whether the correlation between subsequent epidemic characteristics involved in these methods and the epidemic size needs to be further investigated. Quite a few studies have explored the influence of non-drug intervention measures such as lockdown, mask use, and mass-testing on the epidemic of COVID-19 by methods such as the dynamics model [20,21,22,23,24,25,26]. Nevertheless, to further investigate the role of the non-pharmacological interventions, it is also important to examine the impacts of the factors in interventions such as residents’ adherence to lockdown (represented as the new cases on the day of restriction) on the epidemic size in the United States.

We hypothesized that the severity of the COVID-19 epidemic could be characterized by epidemiological indicators in the early stage and subsequent waves of the epidemic. To address this question, we collected publicly available COVID-19 epidemic data from 51 states (including Washington, DC, USA) in the United States. We also conducted the correlation analysis after identifying the epidemic characteristics by the Joinpoint software and multi-logistic fitting. As the high vaccine coverage and delta variant represent different transmission patterns, we limit our data collection and analysis to 31 March 2021. This study aims to identify the impact of the epidemic characteristics in the early stage and subsequent waves on the pre-delta epidemic size in 51 states of the US.

## 2. Results

### 2.1. Spatiotemporal Changes of COVID-19 Pandemic in the United States

The geographic distribution of the COVID-19 epidemic showed a substantial change from 31 March 2020 to 31 March 2021, with a significant shift from the Eastern United States to the Central United States (Figure S1). On 31 March 2020, the incidence in most states was at a relatively low level (<300/100,000), while the highest incidence was reported in Eastern United States (New York, 275/100,000; New Jersey, 125/100,000). By 11 December 2020, there was a substantial increase in incidence in all states across the United States. In particular, there was a strong emergence in the Central United States (North Dakota, 11,445/100,000; South Dakota, 10,136/100,000). From 11 December 2020 to 31 March 2021, the growth of incidence slowed down slightly, but the Central United States remained the most severely affected area.

Remarkably, most of the states that experienced three or four waves were concentrated in the central part of the United States. The Getis–Ord Gi* statistic for total COVID-19 infectious identified Eastern United States as COVID hotspots in early 2020 and the Central United States as hotspots in 2021 and late 2020 (Figure S1). The Anselin’s Local Moran’s I analysis identified New York, located in the Eastern United States, as a high–low outlier in 2020, and “high–high” clusters were mainly found in the Central United States as of 31 March 2021.

### 2.2. Early Epidemic Characteristics of the COVID-19 Epidemic in the US

**Figure 1:**
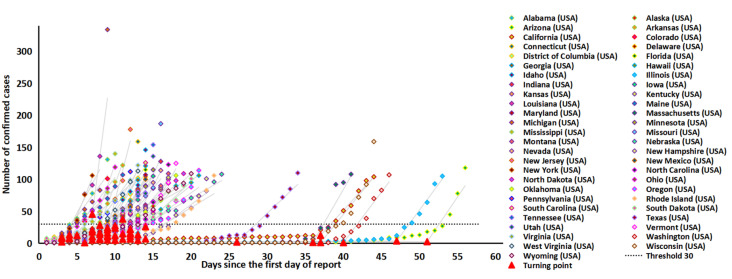
Joinpoint two-phase fitting for 51 U.S. states, showing the transition point below a threshold of 30 cases. Most of the states transited from the slow-growing phase to the fast-growing phase at a level below 30 cases.

We demonstrated two-phase linear fits to the first 100 confirmed cases of COVID-19 during the early phase of the epidemic in the 51 states of the US (Figure 1). The simple model identified one slow-growing phase and one fast-growing phase in the early phase of the 100 confirmed cases. The slow-growing phase was relatively short with a growth rate of 1.6 (1.2–2.0) cases/day, whereas the growth rate in the fast-growing phase was about 11 times higher (18.2 (14.5–21.8) cases/day, Table 1). The conversion from the slow phase to the fast phase occurred on day 13 (9.8–16.1). The average number of confirmed cases at the phase transition point was 12.6 (10.0–15.3) days, and 96.1% (49/51) of the states transited from the slow-growing phase to the fast-growing phase at a level below 30 cases (Figure 1, Table 1). Consistent with a previous finding in China [15], we regarded ‘30’ confirmed cases as a critical threshold where the COVID-19 epidemic started to increase rapidly. Further, in the 51 states, the days required for the number of confirmed cases to increase from 30 to 100 was 5.6 (5.1–6.1) days. The average case–fatality rates in the first 100 confirmed cases across all US states were 1.1% (0.6–1.6%).

### 2.3. Subsequent Epidemic Characteristics Based on Multi-Logistic Fitting

**Figure 2:**
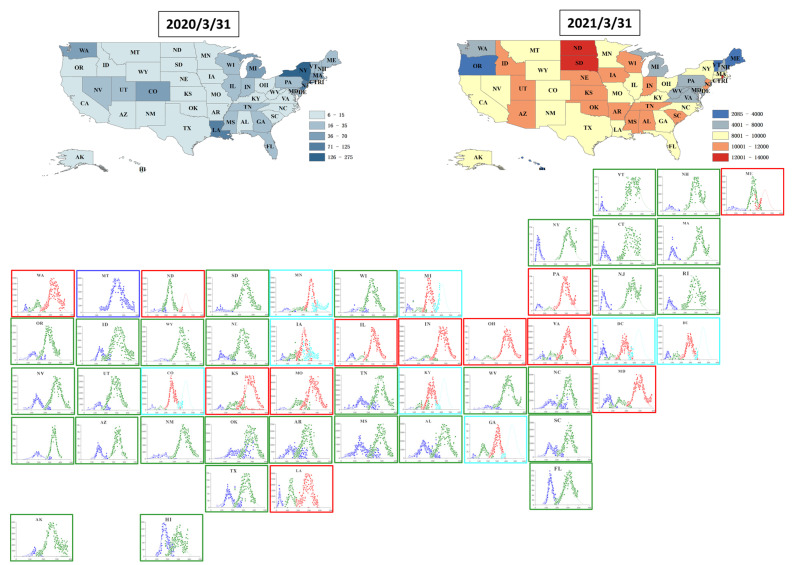
Multi-logistic fitting with multi growth phases by US state. Inset maps display the reported incidence on 31 March 2020 and 31 March 2021. Multi-logistic fittings for the dynamics of the newly reported incidence are presented for each state as separate panels with the blue line, green line, red line and light blue line representing the development process of the first wave, second wave, third wave and fourth wave estimated by fitting, respectively. The types of line colors correspond to the number of waves obtained by fitting. The total number of waves at 1, 2, 3 and 4 were also displayed as the colors of the borders of panels with blue, green, red and bright blue, respectively.

Figure 2 demonstrates the multi-logistic fitting to the COVID-19 epidemics in the 51 states. As of 31st March, our model identified that all 51 states experienced at least 2-waves of COVID-19 growth, among which 23.5% (12/51) experienced a 3-wave growth, and 15.7% (8/51) experienced a 4-wave growth (Table 2). Across all states, the average number of estimated confirmed cases was 111,061 (56,997–165,124), with an outbreak duration of 83.5 (75.0–92.0) days for the first wave. The average number of estimated confirmed cases was 329,686 (178,466–480,907), with an outbreak duration of 96.7 (90.2–103.1) days for the second wave. For the third and fourth waves, the average number of estimated confirmed cases at the peak was 389,757 (256,873–522,641) and 327,861 (43,632–612,091), and the outbreak duration was 94.1 (86.6–101.6) and 92.1 (76.9–107.3) days respectively. The fourth outbreak (estimated to saturate at 327,861 cases), the third outbreak (estimated to saturate at 389,757 cases) and the second outbreak (estimated to saturate at 327,686 cases) is significantly greater than the first outbreak (saturate at 111,061 cases). However, the duration of the outbreaks showed no significant difference across the waves.

### 2.4. Epidemic Size and Associated Characteristics

**Figure 3:**
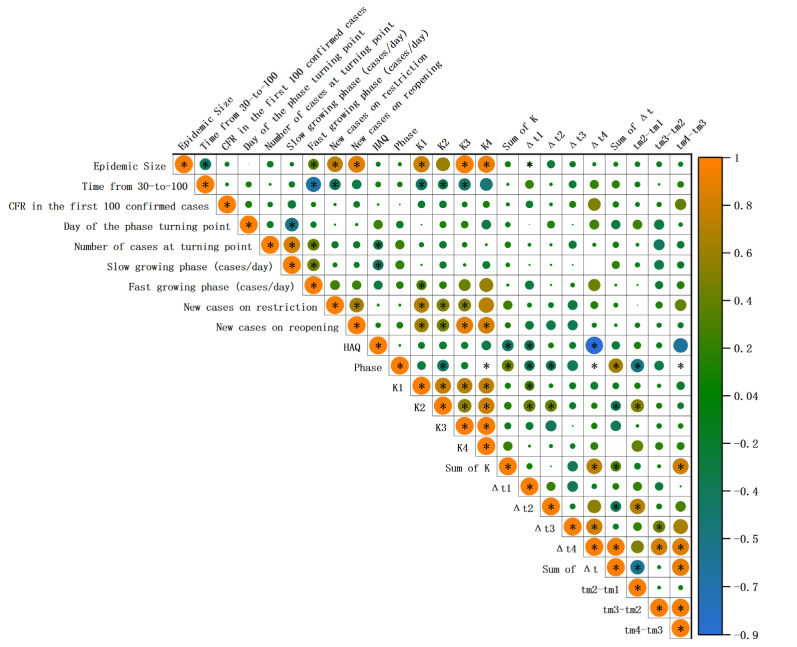
Spearman correlation between epidemic size, multi-logistic parameters, characteristics in the early stage of the epidemic and non-pharmacological intervention characteristics. The size of each circle represents the absolute value of the correlation coefficient. The color of each circle represents the sign of the correlation coefficient and the magnitude of its absolute value. The asterisks in circles indicate that the *p*-value of the hypothesis test for the correlation coefficient is less than 0.05. HAQ: healthcare and access quality index.

As of 31 March 2021, the overall epidemic size was 30,326,324 cases in the United States, ranging from the lowest 19,275 cases in Vermont to 3,669,048 cases in California. Figure 3 demonstrated significant correlations between epidemic size and time from 30 to 100 (*p* = 0.003, r = −0.405) and growth rate of the fast-growing phase (*p* = 0.012, r = 0.351). Additionally, epidemic size also showed significant positive correlations with K_1_ (*p* < 0.001, r = 0.794), K_2_ (*p* < 0.001, r = 0.595), K_3_ (*p* < 0.001, r = 0.977) and K_4_ (*p* = 0.002, r = 0.905). In addition, the epidemic size was positively correlated with the new cases on restriction day (*p* < 0.001, r = 0.764) and new cases on reopening day (*p* < 0.005, r = 0.880).

### 2.5. Correlation between Early and Subsequent Epidemic Characteristics

Notably, early characteristics of the epidemic also show a correlation with overall epidemic-wide fluctuations (Figure 3). Time from 30 to 100 was significantly negatively correlated with the K_1_ (*p* = 0.003, r = −0.41), K_2_ (*p* = 0.028, r = −0.31), and K_3_ (*p* = 0.048, r = −0.45). There is a negative correlation between HAQ and Δt_1_ (*p* = 0.021, r = −0.325) and Δt_4_ (*p* = 0.001, r = −0.929). In contrast, the new cases on reopening day showed a positive correlation with K_1_ (*p* < 0.001, r = 0.618), K_2_ (*p* = 0.003, r = 0.478), K_3_ (*p* < 0.001, r = 0.874), and K_4_ (*p* = 0.042, r = 0.829).

## 3. Discussion

Our study demonstrated that during the early phase of the epidemics in 51 US states, 30 cases appear to be a critical threshold for switching from a relatively slow-growing phase to a fast-growing phase. This is consistent with one of our previous published studies [15]. We identified multiple temporal waves and geographical distribution in the subsequent COVID-19 epidemics. Most states (50/51) have experienced at least 2 waves of the epidemic outbreak. The subsequent waves are significantly stronger and longer than the first wave, but states with a higher first wave tend to have higher subsequent waves as well. We also showed a geographic shift of the epidemic from the coastal states to inland states. The COVID-19 epidemic size across the US states is significantly associated with the duration from 30 to 100 cases, the growth rate of the fast-growing phase, and the peak cases in the subsequent waves.

Our study demonstrated similar early epidemic characteristics in the US compared with the previous findings in China [15]. In both settings, both countries were unprepared for the unprecedented outbreak. Based on the previous study, we again demonstrate that the first 30 cases appear to be an essential indicator of the onset of a rapid phase of COVID-19 transmission. Once this critical level is reached, the epidemic tends to enter a period of rapid expansion. Establishing an early warning system based on the number of confirmed cases per day is crucial to controlling the spread of an epidemic in the early phase. Interventions that aim to contain it to a low level in its early stage may be most beneficial in reducing its subsequent size.

Our study indicated that early characteristics are predictive of the subsequent waves of the epidemics and the epidemic size. Notably, the shorter the duration to increase from 30 to 100 cases in its early phase, the more severe the subsequent epidemic burden. The shorter duration represents a more rapid epidemic spread and likely reflects the absence of effective prevention strategies and diagnostic capacity in the states, leading to a high transmission rate. Consistent with this, more rapid growth in the early phase is also associated with a greater magnitude of subsequent waves. We believe the early rapid development of the outbreak may serve as an important warning signal for the healthcare system to respond to the outbreak and help predict the potential size of COVID-19 in the later development of the epidemic.

Our study demonstrated a significant shift in the geographical distribution of the COVID-19 epidemic from coastal to inland states, and inland states appeared to have experienced more subsequent waves, similar to the results of an earlier study [27]. This might be due to their delay in their response to COVID-19 prevention. California, New Jersey, Illinois, and Connecticut started restrictions early. In comparison, as of 3 May 2020, the eight states in the Central United States had not issued any residence orders nor lockdown interventions. Consistent with this, our study found that the lower the cumulative number of cases on lockdown days, the lower the extent of the subsequent epidemic size. Furthermore, the first eight states that experienced the fourth wave might have higher population density and more frequent changes in social distancing restrictions, such as frequent lockdown and reopening [28,29,30,31,32,33,34]. This may have led to the earlier decline of the previous wave and the earlier appearance of the next wave. Additionally, a low cumulative number of cases on the reopening day also corresponds to a smaller epidemic size intensity. This may also reflect the fact that states that emphasize non-pharmaceutical interventions are more effective at controlling the epidemic [35], which confirmed the view in a previous study that a hasty reopening may lead to another epidemic [10]. However, although many areas would implement restrictions and reopen according to the epidemic situation, the reduced adherence caused by pandemic fatigue may also lead to a large-scale epidemic [36].

Our study also demonstrated a strong correlation between waves. We are the first to identify the number of waves and quantify the size and duration of the waves. We report the subsequent waves were at least three times more than the first waves and each cycle of the waves is about 3 months. The duration likely reflects the time for the healthcare system to react, intervene, and gradually reduce the epidemics.

There are some limitations to our findings. First, our research only verified the pre-delta epidemic data before 31 March 2021 without considering the underestimated cases in the first waves [37] and the effect of the vaccination on the trajectory of the epidemic and multiple rounds of public health interventions. Further investigation is necessary to identify the impacts of these interventions on the course of the COVID-19 epidemic in the US. Second, states in the US may begin to witness the sixth wave of outbreaks with the emergence of another strain. The increasing waves may affect the prediction accuracy of various characteristics of the epidemic. Third, many potential confounding factors such as environmental, meteorological and intervention factors [38,39] were not included in our current study, which demands future investigations. Fourth, the vaccination efficacy, along with the population structure that may influence the vaccine effect and susceptibility [40,41], was not taken into consideration due to the limitation of data sources. The mortality rate was also affected by the complexity of its influencing factors [42,43,44] and was not considered to be an outcome indicator. Moreover, our study only considered the first restriction and first reopening. Many areas have since implemented more restrictions and reopening, which were not included in this study. The number of new cases on the days of other restrictions and reopening would likely serve as important indicators to predict the epidemic size, which could be addressed in further study.

## 4. Materials and Methods

### 4.1. Data Source

We collected publicly available data from 51 states in the United States, that reported on cases of COVID-19 (number of daily confirmed cases, deaths, and recovery cases) from 21 January 2020 to 31 March 2021 from the Coronavirus resource center of Johns Hopkins University and Medicine [3]. In accordance with the methods of another study [45], for some inconsistencies between Johns Hopkins University data and state-level reporting, we have manually supplemented and corrected this dataset and adjusted for reporting-day biases according to the NY Times website [4] to improve the accuracy of our analysis.

We collected social distancing data from the IHME COVID-19 Forecasting Team’s article [46]. We also obtained each state’s population size from the WorldPop Population Counts [47] along with the Healthcare Access and Quality (HAQ) Index, which was a summary measure of personal healthcare access and quality, from the GBD’s article [48].

### 4.2. Selection of Epidemic Characteristics Indicators

We defined the epidemic size for each state as the cumulative number of confirmed cases on 31 March 2021.

For the early epidemic trend of each 51 states, we used the Joinpoint software [49] to identify the trend and transition point of the epidemic during the initial phase of the epidemic based on the first 100 confirmed cases. We imposed a two-phase fit (it can be determined through the Joinpoint software automatically) [15] with the maximum of one joinpoint (corresponding to two-time intervals) and used a linear regression model for both phases. We identified: (1) the time of the transition point between the two phases; (2) the number of cases at the transition point; the growth rates of the (3) first (slow-growing) phase and (4) the second (fast-growing) phase. For the 51 states of the United States, the average number at the turning point is 12.6 (10–15.3) days (Table 1), along with the majority (49/51) of transition points occurring below 30 cases, which is consistent with our previous conclusion of 30 cases in China [15]. Therefore, in this study, we regarded 30 cases as an important threshold for the epidemic growth where the epidemic changed from a slow-growing to a fast-growing phase. We also estimated three additional predictors based on the first 100 confirmed cases, namely: (1) the days required to increase from 30 to 100 cases (time from 30 to 100); (2) the case fatality rate among the first 100 confirmed cases (CFR-100). The ‘first 100 cases’ was defined as the number of confirmed cases on the day the 100th confirmed case was reported.

We also collected various non-pharmacological intervention indicators under the epidemic situation, which were as follows: (1) the time of first restriction and first reopening for 51 US states; (2) the confirmed new cases on the day of restriction and reopening.

For the study period in each of the 51 states, we used a simple multi-logistic fitting (https://logletlab.com/, accessed on 5 April 2022) to identify the key characteristics of the COVID-19 epidemic based on the cumulative number of confirmed COVID-19 cases. We modelled the epidemic patterns by identifying 1 to 4 growth waves of the COVID-19 epidemic. We identified: (1) K_m_ (m = 1,2,3,4) for each wave which represents the asymptotic values that bound the function and therefore specify the level at which the epidemic saturates. The sum of K represents the point at which the epidemic has finally reached saturation; (2) t_m_ for each wave represents the midpoint of each epidemic growth and hence the peak of each outbreak. t_m4_-t_m3_, t_m3_-t_m2_, t_m2_-t_m1_ represent the time interval between the two consecutive outbreaks. The sum of t_m_ represents the sum of the time required for each epidemic to reach its peak; (3) Δt for each phase represents the lengths of time intervals required for the epidemics to grow from 10% to 90% of the saturation level. The sum of Δt represents the sum of the interval lengths required for each epidemic to increase from 10% of saturation level to 90%. Figure S3 is a schematic graph with three main characteristics of K_m_, t_m_ and Δt.

### 4.3. Statistical Analysis

We generated the disease mapping of COVID-19 in ArcGIS10.8 (Environmental Systems Research Institute, Redlands, CA, USA). The reported incidence in each state was joined to the shapefile of state boundary by administrative unit code. We created distribution maps for the COVID-19 on 31 March 2020, 11 December 2020 (the day when the Food and Drug Administration (FDA) of the United States issued an Emergency Use Authorization (EUA) for the first vaccine to prevent COVID-19) [50], and 31 March 2021, separately. We also performed Anselin Local Moran’s I and Getis-Ord Gi* to identify the spatial variations of the COVID-19 in the United States further. For the geospatial analysis, we normalized the epidemic size by dividing the population size of each of the states to obtain the infected rates of per 100,000 individuals.

After verifying the non-normal distribution of most variables through the Kolmogorov–Smirnov test, we used the Spearman correlation test in the OriginPro 2021b software (OriginLab Corporation, Northampton, MA, USA) to examine the correlation between epidemic size, early epidemic characteristics (time from 30 to 100, CFR in the first 100 confirmed cases, day of the phase turning point, number of cases at the turning point, slow-growing phase (case/day), fast-growing phase (case/day)), intervention (new cases on restriction, new cases on reopening) and HAQ indicators and the multi-logistic fitting characteristics of the subsequent epidemics (number of phases; K_1_, K_2_, K_3_, K_4__,_ Sum of K; t_m1_, t_m__2_, t_m3_, t_m4_, sum of t_m_; t_m4_-t_m3_, t_m3_-t_m2_, t_m2_-t_m1_; Δt_1__,_ Δt_2,_ Δt_3_ Δt_4,_ Sum of Δt).

We also compared the differences among K_1_, K_2_, K_3_, K_4_; Δt_1,_ Δt_2,_ Δt_3__,_ Δt_4_ and t_m4_-t_m3_, t_m3_-t_m2_, t_m2_-t_m1_ using the Wilcoxon signed-rank test (GraphPad Prism (Version 8.3.0)).

## 5. Conclusions

In conclusion, we confirmed that the first 30 cases of COVID-19 might be a critical threshold for switching from a relatively slow-growing phase to a fast-growing phase in the early epidemic in the United States. Further, most states have experienced more than 1 wave of the COVID-19 epidemic, and the magnitude of the first wave tends to predict the magnitude of subsequent waves. The pre-delta epidemic size is negatively and significantly correlated with the duration from 30 to 100 cases but positively correlated with the growth rate of the fast-growing phase, and the peak cases in the subsequent 4 waves.

## Figures and Tables

**Table 1:** Key early characteristics in the early-stage of the epidemic and the subsequent size and severity of the epidemic.

State	Number of Confirmed Cases at the Date the 100th Cases were Reported	Number of Deaths at the Date the 100th Cases were Reported	Number of Days from 30 to 100	Case Fatality Rate in the First 100 Confirmed Cases	Day of the Phase Turning Point	Number of Cases at Turning Point	Slow Growing Phase (Cases/Day)	Fast Growing Phase (Cases/Day)
Alabama	106	0	4	0.0%	3	9.73	2.60	16.16
Alaska	102	2	6	2.0%	8	5.80	0.83	8.48
Arizona	104	1	3	1.0%	51	2.60	0.04	17.49
Arkansas	100	0	3	0.0%	8	28.21	3.91	32.41
California	100	0	7	0.0%	37	12.69	0.31	13.04
Colorado	103	1	4	1.0%	5	12.38	2.03	17.03
Connecticut	159	2	4	1.3%	9	10.48	1.38	26.50
Delaware	104	0	6	0.0%	7	10.36	1.68	10.11
District of Columbia	116	2	7	1.7%	9	24.28	2.45	19.18
Florida	109	4	4	3.7%	9	11.49	1.29	16.02
Georgia	118	1	6	0.8%	9	11.79	1.44	19.87
Hawaii	106	0	7	0.0%	12	6.71	0.58	11.25
Idaho	123	0	6	0.0%	8	14.07	1.92	17.73
Illinois	104	0	5	0.0%	47	3.74	0.06	16.14
Indiana	128	4	5	3.1%	12	21.76	2.03	22.60
Iowa	105	0	6	0.0%	10	27.65	2.58	14.21
Kansas	102	2	6	2.0%	9	5.25	0.66	10.37
Kentucky	103	3	5	2.9%	14	26.04	2.09	26.44
Louisiana	103	2	3	1.9%	3	2.17	0.70	22.66
Maine	107	0	7	0.0%	3	7.93	3.58	11.10
Maryland	108	1	5	0.9%	9	16.32	1.85	16.19
Massachusetts	110	0	4	0.0%	36	1.41	0.02	20.75
Michigan	334	3	6	0.9%	7	45.55	7.81	90.68
Minnesota	115	0	6	0.0%	8	7.20	1.04	14.27
Mississippi	140	1	4	0.7%	7	21.09	3.63	34.25
Missouri	130	3	4	2.3%	11	6.56	0.68	25.23
Montana	121	1	6	0.8%	11	14.94	1.53	13.78
Nebraska	102	0	13	0.0%	12	16.23	1.54	6.81
Nevada	165	1	5	0.6%	10	12.34	1.34	14.71
New Hampshire	101	1	6	1.0%	14	7.02	0.48	8.96
New Jersey	176	2	4	1.1%	9	21.22	2.68	46.30
New Mexico	100	0	6	0.0%	11	37.95	3.62	21.07
New York	106	0	3	0.0%	4	13.33	4.38	29.65
North Carolina	104	0	5	0.0%	10	5.14	0.53	11.31
North Dakota	109	2	9	1.8%	6	1.06	0.02	6.50
Ohio	120	0	5	0.0%	4	8.62	2.06	15.74
Oklahoma	106	3	6	2.8%	10	4.78	0.49	10.92
Oregon	114	3	9	2.6%	12	11.16	1.00	8.48
Pennsylvania	101	0	5	0.0%	7	17.83	2.89	16.65
Rhode Island	106	0	6	0.0%	13	4.96	0.33	7.26
South Carolina	126	1	5	0.8%	10	23.82	2.59	19.86
South Dakota	100	1	7	1.0%	12	14.77	0.72	9.47
Tennessee	155	0	6	0.0%	7	5.18	0.81	11.24
Texas	106	1	8	0.9%	26	1.99	0.06	11.27
Utah	112	0	5	0.0%	8	7.16	1.03	14.20
Vermont	123	8	5	6.5%	13	13.52	1.22	20.61
Virginia	115	2	8	1.7%	4	7.25	1.98	10.57
Washington	110	9	5	8.2%	40	1.18	0.01	15.80
West Virginia	113	0	5	0.0%	8	16.71	2.47	19.43
Wisconsin	106	0	4	0.0%	37	1.27	0.01	14.65
Wyoming	121	0	8	0.0%	12	22.14	2.09	10.84
Total/Mean (95%CI)	118.8 (108.9–128.7)	1.3 (0.8–1.8)	5.6 (5.1–6.1)	1.1% (0.6–1.6%)	13 (9.8–16.1)	12.6 (10–15.3)	1.6 (1.2–2)	18.2 (14.5–21.8)

**Table 2:** The fitted parameters for the multi-logistic fitting for the dynamics of the cumulative incidence in each state of US.

State	Phase *	K_1_^1^	Δt_1_^2^	tm_1_^3^	K_2_	Δt_2_	tm_2_	K_3_	Δt_3_	tm_3_	K_4_	Δt_4_	tm_4_	K
Alabama	**2**	158,058	113.0	139.0	356,678	105	287	-	-	-	-	-	-	514,736
Alaska	**2**	4868	74.8	122.0	55,034	113	265	-	-	-	-	-	-	59,902
Arizona	**2**	219,769	76.2	166.0	621,947	82	339	-	-	-	-	-	-	841,716
Arkansas	**2**	89,317	137.0	151.0	250,964	115	289	-	-	-	-	-	-	340,281
California	**2**	937,689	124.0	188.0	2,683,967	67.8	341	-	-	-	-	-	-	3,621,656
Colorado	**4**	27,074	76.9	58.8	29,840	62.8	142	352,188	85.7	273	402,309	86.7	422	811,411
Connecticut	**2**	46,842	54.6	49.9	263,325	124	296	-	-	-	-	-	-	310,167
Delaware	**4**	10,156	50.0	54.8	10,122	78.8	145	71,115	103	294	95,159	92.3	443	186,552
District of Columbia	**4**	10,694	67.2	61.3	4544	87.5	156	257,73	104	296	45,849	113	449	86,860
Florida	**2**	676,134	70.0	141.0	1,417,834	126	309	-	-	-	-	-	-	2,093,968
Georgia	**4**	42,444	54.2	53.9	302,132	97.9	156	651,457	92.6	307	1,051,352	119	469	2,047,385
Hawaii	**2**	14,189	82.8	175.0	15,304	119	302	-	-	-	-	-	-	29,493
Idaho	**2**	31,874	73.2	133.0	147,958	118	266	-	-	-	-	-	-	179,832
Illinois	**3**	129,678	52.8	101.0	122,400	63.3	198	960,542	100	315	-	-	-	1,212,620
Indiana	**3**	39,662	78.6	59.4	65,411	78.1	163	575,259	92.3	278	-	-	-	680,332
Iowa	**4**	23,273	60.0	60.0	52,000	90	150	208,015	70.8	252	65,336	81	326	348,624
Kansas	**3**	16,721	50.0	65.0	43,685	75	170	241,620	96.1	280	-	-	-	302,026
Kentucky	**4**	14,453	64.4	73.3	58,258	87.7	178	331,578	106	288	381,435	93.5	433	785,724
Louisiana	**3**	43,792	30.0	30.0	114,559	58.4	140	289,018	106	292	-	-	-	447,369
Maine	**3**	4594	117.0	86.2	40,123	84.9	296	35,078	107	422	-	-	-	79,795
Maryland	**3**	63,754	65.7	67.0	59,507	78.4	156	279,222	89.6	296	-	-	-	402,483
Massachusetts	**2**	114,764	62.1	91.4	515,403	112	334	-	-	-	-	-	-	630,167
Michigan	**4**	65,674	65.7	45.0	66,908	78.2	157	479,761	77.1	265	480,316	90.8	409	1,092,659
Minnesota	**4**	42,237	60.9	81.5	56,831	69.2	172	304,990	50	259	101,135	60.6	326	505,193
Mississippi	**2**	112,294	140.0	147.0	194,676	96.4	293	-	-	-	-	-	-	306,970
Missouri	**3**	16,177	55.0	50.0	102,000	80	165	399,748	97.2	271	-	-	-	517,925
Montana	**1**	102,982	126.0	253.0	-	-	-	-	-	-	-	-	-	102,982
Nebraska	**2**	26,763	106.0	108.0	179,197	114	283	-	-	-	-	-	-	205,960
Nevada	**2**	79,921	96.3	141.0	221,403	94.9	286	-	-	-	-	-	-	301,324
New Hampshire	**2**	6231	78.4	72.2	76,994	110	306	-	-	-	-	-	-	83,225
New Jersey	**2**	180,120	64.5	49.2	738,290	131	310	-	-	-	-	-	-	918,410
New Mexico	**2**	22,510	108.0	102.0	166,650	101	272	-	-	-	-	-	-	189,160
New York	**2**	411,495	49.5	46.6	1,495,624	121	318	-	-	-	-	-	-	1,907,119
North Carolina	**2**	240,328	146.0	153.0	687,653	104	308	-	-	-	-	-	-	927,981
North Dakota	**3**	9515	109.0	118.0	88,707	83.1	242	46,699	77.4	421	-	-	-	144,921
Ohio	**3**	32,254	58.9	42.0	76,174	50	139	893,327	97.3	281	-	-	-	1,001,755
Oklahoma	**2**	115,630	137.0	169.0	325,333	97.5	296	-	-	-	-	-	-	440,963
Oregon	**2**	27,810	116.0	141.0	135,919	112	294	-	-	-	-	-	-	163,729
Pennsylvania	**3**	82,241	52.0	45.0	44,856	50	144	900,730	110	292	-	-	-	1,027,827
Rhode Island	**2**	17,323	62.2	63.4	118,673	122	295	-	-	-	-	-	-	135,996
South Carolina	**2**	156,103	112.0	145.0	405,559	105	311	-	-	-	-	-	-	561,662
South Dakota	**2**	6409	89.6	58.9	108,453	108	246	-	-	-	-	-	-	114,862
Tennessee	**2**	207,342	126.0	150.0	577,027	95.1	290	-	-	-	-	-	-	784,369
Texas	**2**	786,183	116.0	169.0	2,110,206	122	326	-	-	-	-	-	-	2,896,389
Utah	**2**	46,055	104.0	134.0	346,926	130	289	-	-	-	-	-	-	392,981
Vermont	**2**	1114	41.6	33.9	20,062	153	327	-	-	-	-	-	-	21,176
Virginia	**3**	61,158	88.4	76.7	106,055	91.3	170	454,702	98	308	-	-	-	621,915
Washington	**3**	17,699	47.5	76.0	53,412	76.8	178	294,323	122	331	-	-	-	365,434
West Virginia	**2**	11,017	101.0	130.0	129,068	105	281	-	-	-	-	-	-	140,085
Wisconsin	**2**	56,786	109.0	142.0	568,147	114	288	-	-	-	-	-	-	624,933
Wyoming	**2**	2915	56.0	109.0	52,500	93.7	261	-	-	-	-	-	-	55,415
**Total/Mean (95%CI)**	-	111,060.5 (56,996.6–165,124.4)	83.5 (75–92)	101.5 (87.6–115.5)	329,686 (178,465.5–480,906.5)	96.7 (90.2–103.1)	246.5 (226.9–266.1)	389,757.3 (256,873.4–522,641.1)	94.1 (86.6–101.6)	301.1 (279.8–322.3)	327,861.4 (43,631.9–612,090.8)	92.1 (76.9–107.3)	409.6 (364–455.3)	638,557.2 (434,066.4–843,048.1)

* All the parameters were defined by the multi-logistic fitting; ^1^ The parameters K_1_, K_2_, K_3_, K_4_, and K represent the asymptotic values that bound the function and therefore specify the level at which the epidemic and the overall epidemic saturates; ^2^ The parameters tm_1_, tm_2_, tm_3_, and tm_4_ represent the midpoint of each epidemic growth and hence the peak of each outbreak; ^3^ The parameters Δt_1_, Δt_2_, Δt_3_, and Δt_4_ are the lengths of time intervals required for the epidemics to grow from 10% to 90% of the saturation level.

## Data Availability

The data that support the findings of this study are available from the open data platform of the Coronavirus Resource Center of Johns Hopkins University and Medicine [3], IHME COVID-19 Forecasting Team’s article [46] and WorldPop Population [47].

## Supplementary Materials

The following supporting information can be downloaded at: https://www.mdpi.com/article/10.3390/pathogens11050576/s1, Figure S1: The changes of the geographic distribution of COVID-19 incidence in the United States from 31 March 2020 to 31 March 2021, including 11 December 2020; Figure S2: Comparison of the fitted parameters for the multi-logistic approximation of 50 US states and Washington DC; Figure S3: A schematic graph with three main indicators of K_m_, t_m_ and Δt; Table S1: The Global Moran’s I of COVID-19 incidence in the United States on 31 March 2020, 31 March 2021 and 11 December 2020; Table S2: Spearman correlation between multi-logistic parameters and indicators in the early stage of the epidemic and non-pharmacological intervention indicators.
