# Supplementary material for: What Is the Impact of Early and Subsequent Epidemic Characteristics on the Pre-delta COVID-19 Epidemic Size in the United States?
Source: Pathogens. 2022 May 13;11(5):576. doi: 10.3390/pathogens11050576 (PMC9147779; doi:10.3390/pathogens11050576)
Supplement: Supplementary file 1 [file pathogens-11-00576-s001.zip › pathogens-1692668-supplementary.pdf]

Supplementary Appendix:

This is a supplementary material containing the results of spatiotemporal analysis and Spearman correlation test mentioned in the main text.

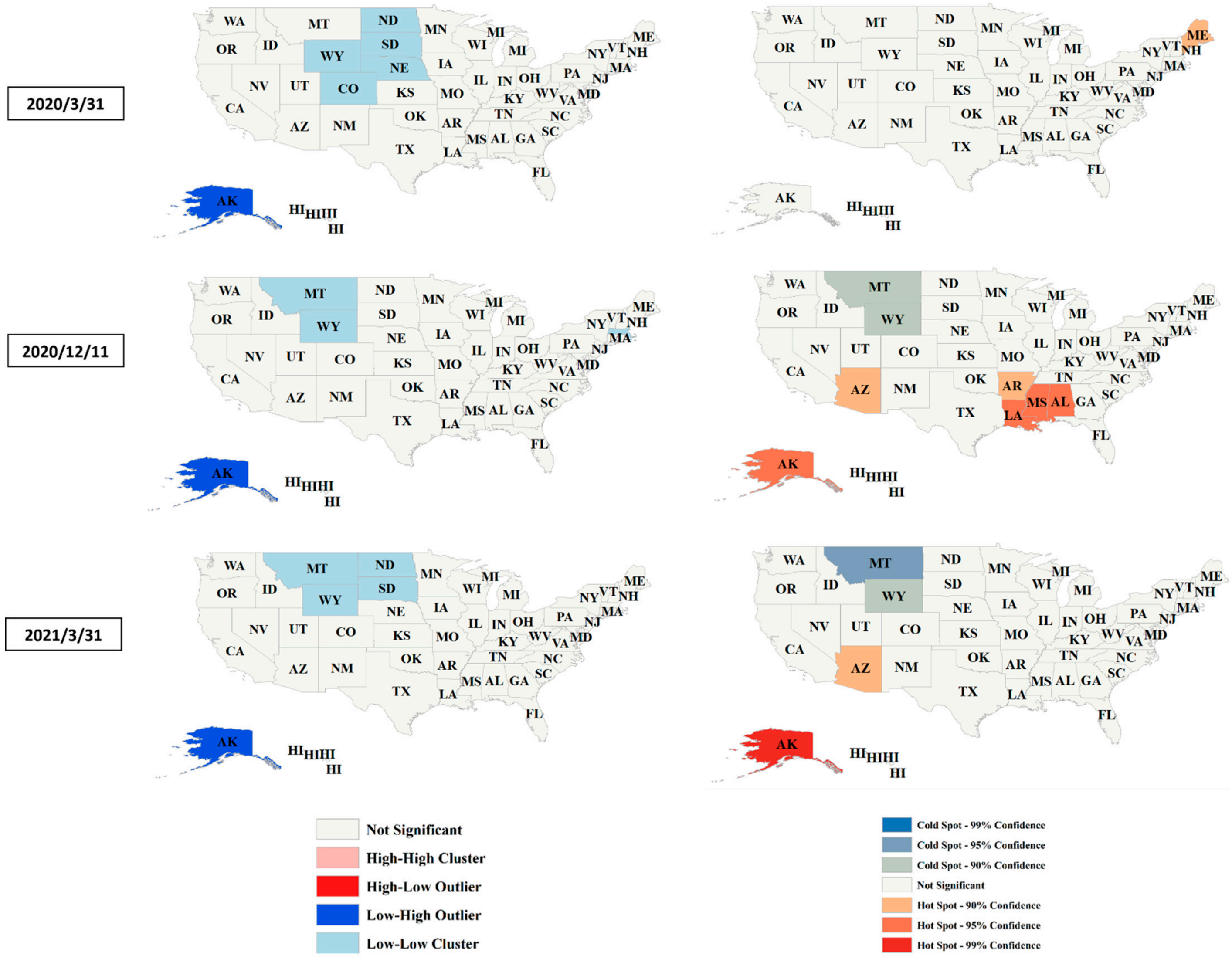

Figure S1. The changes of geographic distribution of COVID-19 incidence in the United States from 31st March 2020 to 31st March 2021, including 11st December 2020.

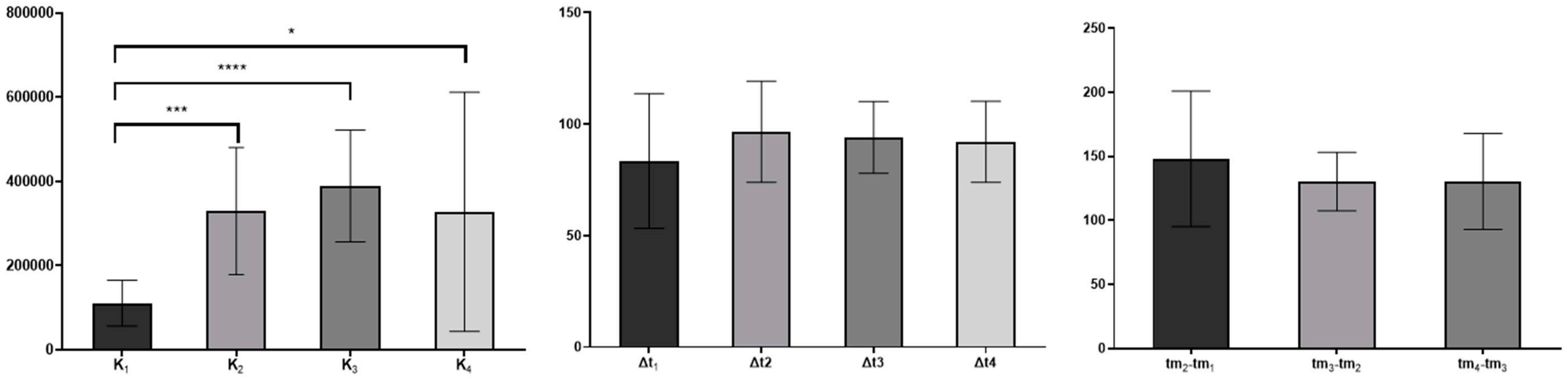

Figure S2. Comparison of the fitted parameters for the multi-logistic approximation of 50 U.S. states and Washington DC. (* represent p<0.05, ** represent p<0.01, * represent p<0.001, **** represent p<0.0001).**

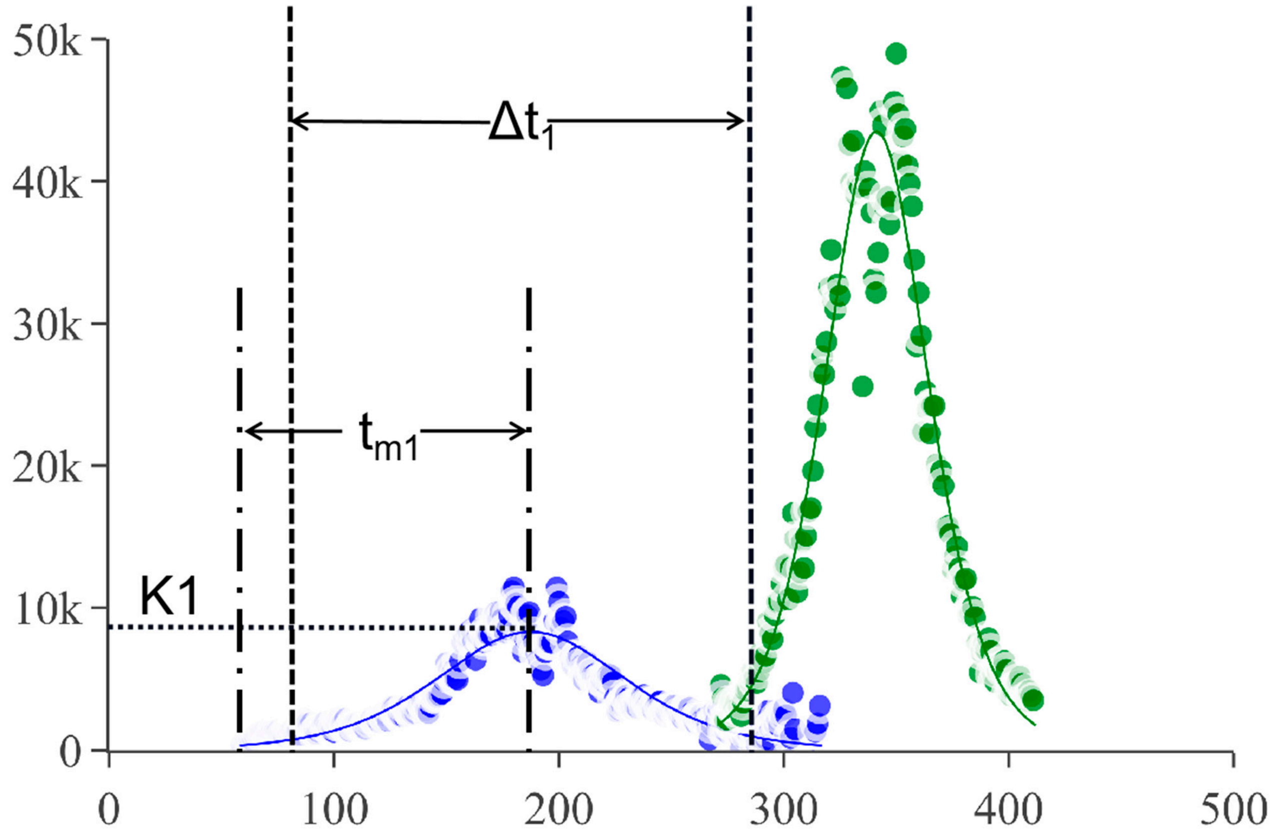

Figure S3. A schematic graph with three main indicators of K_m, t_m and Δt.

Table S1 The Global Moran's I of COVID-19 incidence in the United States on 31st March 2020, 31st March 2021 and 11th December 2020.

Year	Moran's Index	Expected Index	Variance	z-score	*p*-value
2020/3/31	-0.005593	-0.020000	0.000697	0.545635	0.585317
2020/12/11	0.010610	-0.020000	0.002181	0.655389	0.512217
2021/3/31	-0.012263	-0.020000	0.002070	0.170045	0.864975

Table S2. Spearman correlation between multi-logistic parameters and indicators in the early-stage of the epidemic and non-pharmacological intervention indicators. (* represent p<0.05, ** represent p<0.01).

	Epidemic size	Non-pharmacological intervention indicators			Indicators in the early-stage of the epidemic					
		New cases on restriction	New cases on reopening	HAQ	Day of the phase turning point	Number of cases at turning point	Slow growing phase (cases/day)	Fast growing phase (cases/day)	Time from 30-to-100	Case fatality rate in the first 100 confirmed cases
**Phase**	0.047	0.027	0.138	-0.020	-0.172	0.261	0.244	0.171	0.088	0.008
**K1**	**0.794****	**0.694****	**0.618****	-0.148	-0.009	-0.082	-0.014	**0.305***	**-0.406****	-0.173
**K2**	**0.595****	**0.505****	**0.478****	-0.182	0.148	-0.173	-0.163	0.174	**-0.311***	-0.174
**K3**	**0.977****	**0.506***	**0.874****	-0.162	0.175	0.092	0.035	0.421	**-0.447***	-0.100
**K4**	**0.905****	0.714	**0.829***	-0.286	-0.277	0.024	-0.190	0.595	-0.491	0.146
**SUM_K**	0.108	0.262	0.116	**-0.401****	-0.094	0.118	0.067	0.029	-0.029	-0.047
**Δt_1**	-0.048	-0.074	-0.233	**-0.325***	-0.003	0.040	0.013	-0.248	0.229	-0.178
**Δt_2**	-0.200	-0.097	-0.275	0.102	0.180	0.027	-0.014	-0.014	-0.035	-0.041
**Δt_3**	-0.118	-0.290	-0.316	-0.148	-0.007	-0.201	0.010	0.066	-0.185	0.148
**Δt_4**	-0.048	0.179	-0.086	**-0.929****	0.289	0.048	0.000	0.452	0.245	0.512
**Sum of Δt**	0.073	0.077	0.036	-0.221	-0.261	0.118	0.198	0.027	0.193	0.127
**tm1**	-0.012	-0.026	-0.174	-0.180	**0.277***	-0.193	**-0.284***	**-0.314***	0.177	-0.215
**tm2**	0.070	0.111	-0.002	0.214	**0.383****	-0.190	-0.244	-0.024	-0.095	-0.079
**tm3**	-0.204	-0.006	-0.178	0.130	-0.113	**-0.451***	-0.281	-0.346	0.331	0.036
**tm4**	-0.108	0.179	-0.143	**-0.766***	0.085	-0.216	-0.216	0.228	0.220	0.393
